# Carbon isotope fractionation reveals distinct process of CH_4_ emission from different compartments of paddy ecosystem

**DOI:** 10.1038/srep27065

**Published:** 2016-06-02

**Authors:** Guangbin Zhang, Haiyang Yu, Xianfang Fan, Jing Ma, Hua Xu

**Affiliations:** 1 State Key Laboratory of Soil and Sustainable Agriculture, Institute of Soil Science, Chinese Academy of Sciences, Nanjing 210008, China; 2 University of Chinese Academy of Sciences, Beijing 100049, China

## Abstract

Carbon isotopic fractionations in the processes of CH_4_ emission from paddy field remain poorly understood. The δ^13^C-values of CH_4_ in association with production, oxidation and transport of CH_4_ in different pools of a paddy field were determined, and the stable carbon isotope fractionations were calibrated to assess relative contribution of acetate to CH_4_ production (*f*_ac_) and fraction of CH_4_ oxidized (*f*_ox_) by different pathways. The apparent isotope fractionation for CO_2_ conversion to CH_4_ (*α*_app_) was 1.041–1.056 in the soil and 1.046–1.080 on the roots, indicating that *f*_ac_ was 10–60% and 0–50%, respectively. Isotope fractionation associated with CH_4_ oxidation (*α*_ox_) was 1.021 ± 0.007 in the soil and 1.013 ± 0.005 on the roots, and the transport fractionation (*ε*_transport_) by rice plants was estimated to be −16.7‰ ~ −11.1‰. Rhizospheric *f*_ox_ was about 30–100%, and it was more important at the beginning but decreased fast towards the end of season. Large value of *f*_ox_ was also observed at the soil-water interface and soil and roots surfaces, respectively. The results demonstrate that carbon isotopic fractionations which might be different in different conditions were sensitive to the estimations of *f*_ac_ and *f*_ox_ in paddy field.

Methane (CH_4_) is the second most important greenhouse gas after carbon dioxide (CO_2_). On a 100-year horizon, CH_4_ has 25 times the global warming potential of CO_2_. Paddy fields are one of the largest anthropogenic sources of atmospheric CH_4_, contributing to 33–40 Tg yr^−1^ during the 2000–2009^1^. The global CH_4_ emission from paddy fields will continually increase by intensification of rice cultivation and expansion of planting area to meet the demands of the growing populations^2^,^3^,^4^. Paddy CH_4_ emission is an integrated effect of the production, oxidation and transport of CH_4_ in the field. A better knowledge of these processes affecting CH_4_ emission may provide more information for effectively mitigating CH_4_ emission in agricultural ecosystems.

The technique of stable carbon isotopes has been proved to be a useful tool in studying the processes of CH_4_ emission^5^,^6^,^7^,^8^,^9^. Isotope fractionation happens in all the major processes CH_4_ emission, namely, ^12^C-substrate is preferentially utilized by methanogens for CH_4_ production, and once formed, ^12^CH_4_ is consumed faster than ^13^CH_4_ by methanotrophs, and ^12^CH_4_ is transported faster than ^13^CH_4_ as well^10^,^11^,^12^. Thereby, measurements of the δ^13^C in production, oxidation and transport of the CH_4_ from different pools of the field are benefical to support a process-based model for CH_4_ emission^8^,^13^,^14^. Moreover, the relative contribution of acetate to CH_4_ production (*f*_ac_) and the fraction of CH_4_ oxidized (*f*_ox_) can be quantitatively estimated^5^,^6^,^7^ using mass balance equations based on the measurements of δ^13^C in CH_4_, CO_2_ and acetate, and of the isotope fractionation factors (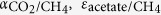, *α*_ox_ and *ε*_transport_). Investigations on 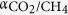, 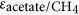 and *ε*_transport_ of paddy fields were carried out greatly^5^,^15^,^16^,^17^,^18^, however, few data are available on *α*_ox_ for CH_4_ oxidation by methanotrophs in paddy soils, in particular *α*_ox_ on rice roots^19^.

Some uncertainties also exist in the δ^13^CH_4_ that are used as newly produced δ^13^CH_4_ (δ^13^CH_4 (original)_) and finally oxidized δ^13^CH_4_ (δ^13^CH_4 (oxidized)_) in different studies for quantifing *f*_ac_ and *f*_ox_. For example, former reports in USA using porewater δ^13^CH_4_ as δ^13^CH_4 (original)_^6^,^7^ whereas anaerobically produced δ^13^CH_4_ was used in Italy^5^,^20^ and China^19^,^21^. They believed that porewater CH_4_ was a poor proxy for δ^13^CH_4 (original)_ as it was potentially affected by CH_4_ oxidization and transport in field conditions^11^,^22^. Similarly, various δ^13^CH_4_, such as rhizospheric δ^13^CH_4_, aerobically produced δ^13^CH_4_, porewater δ^13^CH_4_ or floodwater δ^13^CH_4_ sometimes, have been regarded as δ^13^CH_4 (oxidized)_ for estimation of *f*_ox_ in the rhizosphere or at the soil-water interface^5^,^6^,^7^,^9^,^19^,^23^. More importantly, large differences were observed in the estimated *f*_ac_ and *f*_ox_ if different δ^13^CH_4 (original)_ and δ^13^CH_4 (oxidized)_ was assumed in the same study^5^,^7^. Therefore, more comparable observations in different conditions with corresponding isotope fractionation factors are needed to discuss δ^13^CH_4 (original)_ and δ^13^CH_4 (oxidized)_ in accurate estimations of *f*_ac_ and *f*_ox_.

In this study, field and incubation experiments were conducted to observe the process of CH_4_ emission closely related to the production, oxidation and transport of CH_4_, including CH_4_ fluxes emitted from the field and via the plants, CH_4_ concentrations in the aerenchyma of the plants, and in soil pore water and floodwater, CH_4_ production and oxidation rates in the soil and on the roots, and all the corresponding δ^13^CH_4_. The objectives of the present study were (1) to improve our understanding of the processes in CH_4_ emission by measurements of the stable carbon isotopes, (2) to investigate the isotope fractionation factor *α*_ox_ in the soil and on the roots, and (3) to discuss the availabilities in the estimation of *f*_ac_ and *f*_ox_ associated with different pools of δ^13^CH_4_ in the field.

## Results

### CH_4_ production and δ^13^C of CH_4_ and CO_2_

**Figure 1:**
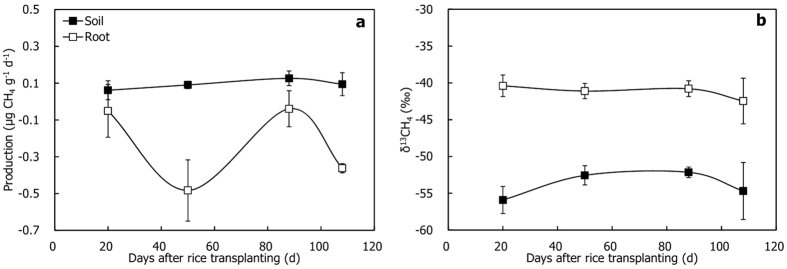
Temporal variations of CH_4_ production rates in the soil and on the roots under aerobic (**a**,**b**) incubation, and corresponding δ^13^CH_4_.

In anaerobic incubation, both CH_4_ production potentials in the soil and on the roots were relatively low on 20 days after rice transplanting (D20), peaked (2.2 *μ*g CH_4_ g soil^−1^ d^−1^ and 11.1 *μ*g CH_4_ g root^−1^ d^−1^) on D50, and then turned downwards gradually to the bottom on D108 (Table 1). For δ^13^C-value of the produced CH_4_, it was more and more positive in the soil during the whole observational period, being from −71.1‰ to −53.9‰ (Table 1). For CH_4_ produced on the roots however, it was most ^13^C-depleted on D50 and then ^13^C-enriched again on D88, with δ^13^C-value ranging between −86.9‰ and −66.6‰ (Table 1). Throughout the whole season, δ^13^C-value of produced CO_2_ increased from −18.8‰ to −14.9‰ in the soil while decreased from −15.0‰ to −23.3‰ on the roots (Table 1). CH_4_ production under aerobic incubation was hardly observed in the soil (0.06 to 0.13 *μ*g CH_4_ g soil^−1^ d^−1^), particularly on the roots, which was lower than 0 *μ*g CH_4_ g root^−1^ d^−1^ over the whole season (Fig. 1a). The δ^13^C-value of CH_4_ produced in the soil was relatively stable around −55‰ while on the roots it was about −40‰ (Fig. 1b). Apparently, the δ^13^C-values of CH_4_ produced in aerobic incubation were significantly higher (*P* < 0.05) than those of the CH_4_ that was produced in anaerobic incubation (Table 1 and Fig. 1).

### CH_4_ concentration and δ^13^C of CH_4_ and CO_2_

**Figure 2:**
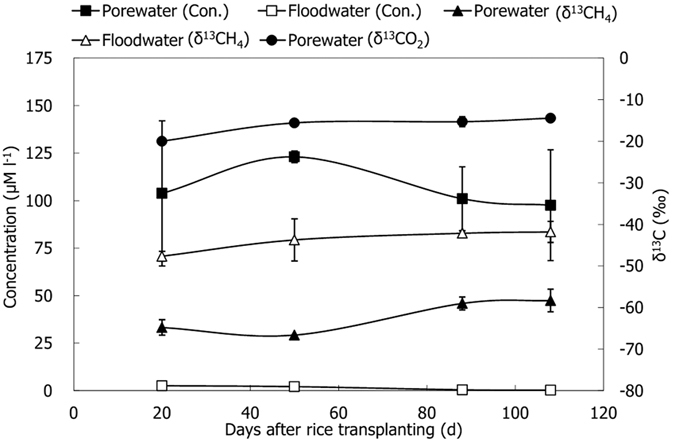
Temporal variations of CH_4_ concentrations in soil pore water and floodwater, and corresponding δ^13^C-values of CH_4_ and CO_2_.

CH_4_ concentration in soil pore water was more than 100 *μ*M L^−1^ in most part of the season (Fig. 2), and it was highest (~120 *μ*M L^−1^) on D50. CH_4_ concentration in floodwater was in the range of 0.21–2.6 *μ*M L^−1^, being significantly lower than that of soil pore water over the season (*P* < 0.01). The δ^13^C-values of CH_4_ in soil pore water and floodwater appeared to increase simultaneously (Fig. 2), from ~−70‰ to −60‰ and from ~−50‰ to −40‰, respectively. Obviously, CH_4_ in soil pore water was more depleted in ^13^C than that of floodwater CH_4_ (*P* < 0.05), indicating that porewater CH_4_ was intensively affected by CH_4_ oxidation at the soil-water interface when it released into the atmosphere. CO_2_ in soil pore water tended to ^13^C-enriched gradually during the rice season, with δ^13^C-values ranging from −20.0‰ to −14.5‰ (Fig. 2).

### CH_4_ oxidation and δ^13^C of CH_4_

CH_4_ oxidation potential in the soil peaked on D50 (6.9 *μ*g CH_4_ g soil^−1^ d^−1^), and then it dropped gradually to the lowest on D108 (Table 2). In contrast, it was highest on the roots (580 *μ*g CH_4_ g root^−1^ d^−1^) on D20 and decreased sharply on D50. After an increase on D88, it decreased again to the lowest on D108 (Table 2). The δ^13^C-values of CH_4_ before oxidization were −41.0‰ ~ −38.4‰ in the soil and −40.5‰ ~ −36.0‰ on the roots. After CH_4_ oxidization, the CH_4_ both in the soil and on the roots were more enriched in ^13^C, with δ^13^C-values of −38.4‰ ~ −32.5‰ and −34.0‰ ~ −26.5‰, respectively (Table 2).

### Plants emitted and aerenchymatic CH_4_

On the three sampling days (D37, D62 and D98) during the season, CH_4_ emitted via the plants was relatively stable with δ^13^C-values of −63.9‰, −62.6‰ and −63.5‰, respectively. For δ^13^C-values of aerenchymatic CH_4_, they were −49.2‰, −45.9‰ and −52.4‰, respectively, being significantly higher in comparison of the emitted CH_4_ (*P* < 0.05). As a result, the isotope fractionations due to CH_4_ transport (*ε*_transport_) were measured to be −14.7‰, −16.7‰ and −11.1‰, respectively, with a mean value of −14.2‰.

### CH_4_ emission and δ^13^C of CH_4_

**Figure 3:**
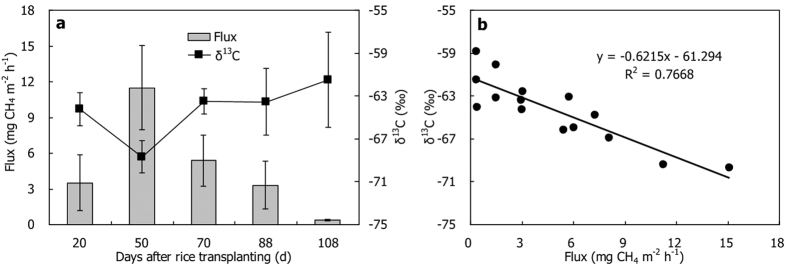
Temporal variations of CH_4_ flux and δ^13^CH_4_ (**a**) and they relationship (**b**).

The CH_4_ flux varied significantly, with the highest value appeared on D50 and the lowest on D108, ranging from 0.4 to 11.5 mg CH_4_ m^−2^ h^−1^ during the observational period (Fig. 3a). The δ^13^CH_4 (emission)_ varied between −68.7‰ and −61.5‰ with the variation pattern just opposite to that of CH_4_ flux (Fig. 3a). It is noteworthy that a significant negative relationship between CH_4_ flux and corresponding δ^13^CH_4 (emission)_ was observed (Fig. 3b). Soil temperature ranged from 17.2 °C to 30.5 °C during the rice season, with a value of 24.5 °C on average.

### δ^13^C of organic carbon in soil and plant samples

The values of δ^13^C in soil organic carbon did not show much variation during the rice season, being −26.84‰ on D37 and −27.66‰ on D108, respectively. The organic carbon in the plant samples also remained relatively stable over the season, with δ^13^C-values being −29.19‰ on D37 and −28.70‰ on D108, respectively, although they were slightly lighter than those of the soil organic carbon.

## Discussion

### CH_4_ production

**Figure 4:**
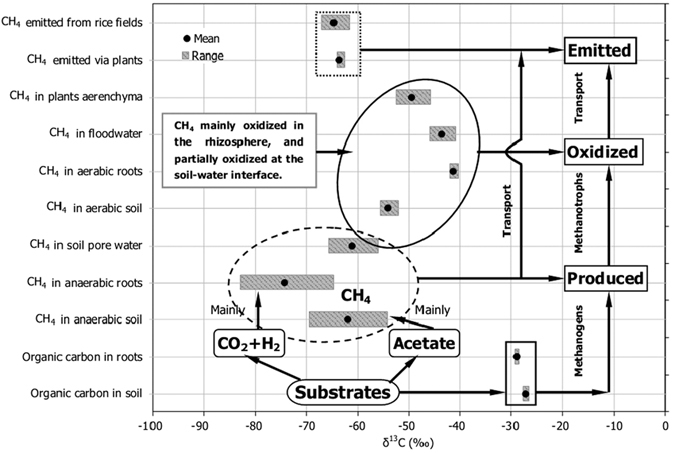
Stable carbon isotopes in the processes of CH_4_ emission from the paddy field. Note: each δ^13^C-value was given in arithmetic mean of the rice season.

The processes of CH_4_ production, oxidation, transport and emission from paddy field were well presented by the measurements of stable carbon isotopes in CH_4_ from different pools of the field (Fig. 4). The decomposition of plants debris and root exudates, besides soil organic matters in the bulk soil, is very important to methanogenesis in paddy field^24^. As a key precursor for methanogens, it was slight ^13^C-depletion on the roots relative to soil organic carbon (Fig. 4). Previous studies also showed δ^13^C-value of organic carbon relatively negative in the plant than in the soil^5^,^19^. Paddy field CH_4_ is mainly produced out of either cleavage of acetate (*f*_ac_) or reduction of H_2_/CO_2_ (1 − *f*_ac_), and the δ^13^C-value of produced CH_4_ primarily depends on relative contribution of the two main methanogenic pathways^10^,^25^. The *f*_ac_ was calculated by the following mass balance^6^,^7^:


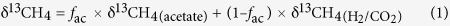


During the process of acetate fermentation forming CH_4_, isotopic fractionation occurs and the fractionation factor is generally expressed to 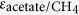. It was found to be −21‰ in pure cultures of acetoclastic *Methanosarcina barkeri*^26^ and −18‰ for acetoclastic *Methanosaeta concilii*^15^,^27^. Using 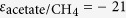
‰, Krüger *et al*.^5^ estimated δ^13^C of CH_4_ produced from acetate (δ^13^CH_4 (acetate)_) between −43‰ and −37‰ according to the measurements of δ^13^C_acetate_ (−22‰ ~ −16‰) in soil pore water of an Italian rice field. Meanwhile, both values of −43‰ and −37‰ have well been applied in many studies^6^,^7^,^9^,^16^,^19^,^21^. Due to a lack of knowledge on 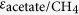 and in order to compare the data interpretation with those of above mentioned, both δ^13^CH_4 (acetate)_ of −43‰ and −37‰ were used in the present study (Table 3).

When H_2_/CO_2_ reduction produces CH_4_, isotopic fractionation factor 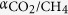 is defined by Hayes^28^:


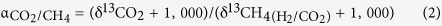


where 
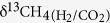
 is δ^13^C of the CH_4_ produced from H_2_/CO_2_ reduction. In addition, based on the ratio of δ^13^CO_2_ to δ^13^CH_4_ in anaerobic incubation (Table 1), an approximation of apparent fractionation (*α*_app_) between CO_2_ and CH_4_ can be calculated by using *α*_app_ = (δ^13^CO_2_ + 1000)/(δ^13^CH_4_ + 1000). The *α*_app_ is calculated from the isotopic signatures of total CH_4_ produced from H_2_/CO_2_ reduction and acetate cleavage, and theoretically, it is lower than 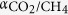. Results of 16 different lake sediments from tropical freshwater wetlands in Brazil^29^ have well demonstrated that 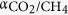 (1.075 ± 0.008) is much higher than *α*_app_ (1.059 ± 0.009). In this study (Table 1), the *α*_app_ decreased gradually from 1.056 on D20 to 1.041 on D108 for the soil. In contrast, it increased sharply from 1.058 on D20 to 1.080 on D50, and then decreased again to 1.053 for the roots. Totally, *α*_app_ was relatively lower in the soil (1.041–1.056) than on the roots (1.046–1.080). The 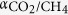 was hence assumed to be 1.050–1.060 in the soil and 1.070–1.080 on the roots (Table 3). Incubating three different soils, Conrad *et al*.^17^ also found that 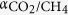 was between 1.050 and 1.060 for two paddy soils. Additionally, previous studies approved the relatively larger 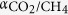 (≥1.070) on the roots than in the soil due to their methanogenic populations were different^16^,^30^.

The CH_4_ produced in anaerobic incubation changed significantly during the rice season, and it was much more ^13^C-enriched in the soil than on the roots (Table 1). It indicates that methanogenic pathway was changed with rice growing, and also demonstrates that acetate-dependant methanogenesis was more important in the soil. In this study, *f*_ac_ in the soil was initially very low (<10%) on D20, but it increased obviously with the rice growing and reached over 60% on D108. In contrast, *f*_ac_ on the roots was relatively high (~30–40%) on D20. It decreased sharply in the middle of the season (near 0%) and then increased again to about 50% on D108. As a whole, *f*_ac_ was relatively higher in the soil than that on the roots (Table 3). Previous study in Italian paddy soil has also demonstrated that acetoclastic methanogenesis was higher than 60% at the end of the season^5^. High contribution of H_2_/CO_2_-dependent methanogenesis to total CH_4_ production on rice roots was considerably reported^5^,^9^,^16^,^19^, and the major reasons were supposed to be the methanogens population on rice roots dominated by Rice Cluster I archaea^31^,^32^,^33^. Methanogenic substrates of organic carbon in the plant appeared to be slightly ^13^C-depleted relative to those of the bulk soil (Fig. 4), which might be a potential reason for the lower *f*_ac_ in the soil.

On the other hand, Belik *et al*. and Tyler *et al*.^6^,^7^ estimated *f*_ac_ of the USA paddy fields by using porewater δ^13^CH_4_ as δ^13^C-value of the produced CH_4_, and they found that it was as high as 80% when 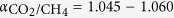. However, Canadian field data have showed that porewater CH_4_ is possibly influenced by CH_4_ oxidation and transport^22^, and in an Italian paddy field Krüger *et al*.^5^ also considered that porewater CH_4_ was a poor proxy for produced CH_4_ due to the potential CH_4_ oxidation therein. Recently, a pot experiment in Germany suggested that porewater CH_4_ could be used as newly produced CH_4_ after tillering stage since they were similar in δ^13^C^34^. In this study, both porewater δ^13^CH_4_ and produced δ^13^CH_4_ generally tended to be enhanced during the rice season (Table 1 and Fig. 2), and on average they were similar with each other (Fig. 4). According to δ^13^C-values of porewater CH_4_ and CO_2_ (Fig. 2), it was found that the *α*_app_ in soil pore water was from 1.047 to 1.054. Therefore, 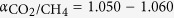 was assumed for comparing with former reports^6^,^7^ and present data of the paddy soil. Hydrogenotrophic methanogenesis was estimated to be dominated over the season (~60–80%), which differed much from the field data in USA^6^,^7^. More importantly, the methanogenic pathway in soil pore water was different from that in paddy soil (Table 3). Although reasons for the difference in *f*_ac_ between paddy soil and porewater were not clear, it is not recommended here that porewater CH_4_ was absolutely regarded as newly produced CH_4_.

### CH_4_ oxidation

The produced CH_4_ in paddy field is mainly oxidized in the rhizosphere and at the soil-water interface, and accurate estimation of the CH_4_ oxidation is one of the major aims of this study. Compared to δ^13^C of newly produced CH_4_ (δ^13^CH_4 (original)_), δ^13^C of remaining CH_4_ after it has undergone oxidization (δ^13^CH_4 (oxidized)_) was significant ^13^C-enriched (Fig. 4). Therefore, fraction of the CH_4_ that is oxidized (*f*_ox_) in the field can be estimated by the mass balance equation^6^,^7^:


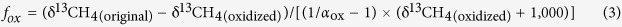


In general, anaerobically produced δ^13^CH_4_ is regarded as δ^13^CH_4 (original)_ and δ^13^CH_4 (oxidized)_ is estimated by the measurements of δ^13^CH_4 (emission)_ corrected with transport fractionation factor (*ε*_transport_) using a semi-empirical equation^5^,^7^,^16^:


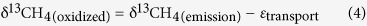


In the closed-system incubation, CH_4_ oxidation fractionation factor *α*_ox_ is known to be calculated according to the Rayleigh equation^35^,^36^:


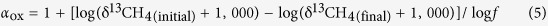


where δ^13^CH_4 (initial)_ stands for δ^13^C-value of CH_4_ at time 0, δ^13^CH_4 (final)_ for δ^13^C-value of CH_4_ at time t, and *f* (%) for the percentage of CH_4_ remaining at time t.

To our knowledge, *α*_ox_ = 1.025 − 1.038 at a temperature of 12–35 °C is initially measured in methanotrophs-enriched cultures^35^ and then widely in landfill cover soils^36^,^37^,^38^, and it has substantially been used in the studies of paddy soil^5^,^6^,^7^,^9^,^20^,^21^,^23^. Recently, *α*_ox_ = 1.025 − 1.033 was found in a Chinese paddy soil at 28.3 °C^19^. By far, reports on *α*_ox_ in paddy soil, in particular on rice roots, are very few available. In the present study (Table 2), *α*_ox_ in the soil firstly increased from 1.014 on D20 to the highest value of 1.030 on D88, and then it decreased again to 1.021 on D108. In contrast, *α*_ox_ on the roots generally declined from 1.019 on D20 to the lowest 1.008 on D108. As a whole, it was higher in the soil (1.021 ± 0.007) than on the roots (1.013 ± 0.005) at 24.5 °C, being much lower than those of measured or used in previous studies under a similar temperature as above mentioned. In addition to *α*_ox_-value of 1.021 ± 0.007 in the soil and 1.013 ± 0.005 on the roots was used, we made an alternative calculation using *α*_ox_ = 1.038 for better comparable to the previous studies (Table 4). Reasons for the difference in *α*_ox_ between paddy soils and rice roots are not understood, but Jahnke *et al*.^39^ found that there were complex factors influencing the isotopic fractionation in CH_4_ oxidation and carbon assimilation in various methanotrophs. Besides, main groups of methanotrophs in rice microcosm (*Methylococcaceae* and *Methylocystaceae*) are active, but their dominance may change depending upon substrate supply and nutrient status^40^,^41^. Therefore, it is no wonder that our measurement of *α*_ox_ in paddy soil was different from that on rice roots, and that both of them differed much from that found in different environments and habitats as above mentioned. On the other hand, *ε*_transport_ is equivalent to the difference between emitted and aerenchymatic δ^13^CH_4_^5^,^7^, and it was estimated to be −14.2‰ on average (Detailed descriptions please see below).

Rhizospheric CH_4_ oxidation was the most important on D20 (Table 4). At that time, almost the produced CH_4_ was oxidized before it was emitted into the atmosphere. With the rice growing, the *f*_ox_ decreased fast to ~30% in the end. Both CH_4_ oxidation potentials in the soil and on the roots were highest between D20 and D50, and decreased gradually towards the end of the season (Table 2), which might be the important reason. *In situ* inhibitor experiments, Krüger *et al*.^42^ also found that *f*_ox_ was highest just at the beginning of the season with a peak of ~40%, and then it became negligible at the end of the season. Soon later, it was reported that *f*_ox_ was no more than 50% over the season and it decreased rapidly from the beginning till the end of the season^5^,^20^. They further concluded the possible reason was that activities of the methanotrophs were limited by nitrogen consumption with the rice growing under field conditions^5^,^20^,^42^.

When porewater CH_4_ released into the floodwater of the paddy fields, it was strongly oxidized at the soil-water interface since floodwater CH_4_ was much more ^13^C-enriched than porewater CH_4_ (Fig. 2). So, *f*_ox_ in this oxidizing area, in principle, can be estimated using porewater δ^13^CH_4_ as δ^13^CH_4 (original)_ and floodwater δ^13^CH_4_ as δ^13^CH_4 (oxidized)_. Value of *f*_ox_ was found to be over 80% throughout the whole season, which was generally higher than that of *f*_ox_ in the rhizosphere (Table 4). Although *f*_ox_ at the soil-water interface appeared to be much high, the amount of the CH_4_ oxidized must be significantly lower than that in the rhizosphere. Because produced CH_4_ is mostly oxidized in the rhizosphere during the rice-growing season as over 90% of the CH_4_ emits to the atmosphere through the aerenchyma of the plants while less than 0.1% releases via ebullition and diffusion^24^,^43^,^44^. In addition, it was reported that the absolute CH_4_ oxidation rate at the soil-water interface was much lower than that in the rhizosphere^24^,^45^,^46^.

Compared to methanogenesis in anaerobic soil, that was in aerobic soil significantly lower in CH_4_ production rate but more positive in δ^13^C (Fig. 4). The findings demonstrate that intensive CH_4_ oxidization happened at the soil surface in lab conditions. As a result, *f*_ox_ at the soil surface (Table 4) was estimated using anaerobically produced δ^13^CH_4_ as δ^13^CH_4 (original)_ and aerobically produced δ^13^CH_4_ as δ^13^CH_4 (oxidized)_^19^. It was the highest (~80%) at the beginning of the season and decreased rapidly later (<0%). In field conditions, CH_4_ oxidation in paddy field without rice plants occurs mainly at the soil-water interface, which is similar to CH_4_ oxidation under aerobic incubation in lab conditions. Therefore, it is feasible to quantitatively estimate *f*_ox_ in paddy fields during the non-rice-growing season or at the soil-water interface during the rice-growing season based on the difference in δ^13^CH_4_ between anaerobic and aerobic incubations. What is more, *f*_ox_ at the root surface was also estimated by comparing δ^13^C-value of the CH_4_ produced under aerobic conditions with those under anaerobic conditions (Table 4). It was found that *f*_ox_ at the root surface stayed over 100% throughout the whole season. Even if the *α*_ox_ = 1.038 was used, it was still as high as 100% (Table 4), further suggesting that CH_4_ oxidation on rice roots was extremely strong indeed. CH_4_ oxidation rate much higher on the roots (Table 2) was supposed to be the main reason for the *f*_ox_ was higher than that in the soil.

### CH_4_ transport and emission

Transporting CH_4_ is the last step of CH_4_ emission from paddy field to the atmosphere. Although CH_4_ oxidation leads to the produced CH_4_ obviously enriched in ^13^C, isotope fractionation in CH_4_ transport offsets the positive effect on δ^13^CH_4_, causing the CH_4_^13^C-depleted again^13^,^22^. As a result, the δ^13^C-values of emitted CH_4_ were close to the produced δ^13^CH_4_ (Fig. 4). The isotope fractionation changes with the efficiency of CH_4_ transport in growth of the plants^5^,^9^,^23^. In the middle of the season, CH_4_ transport capacity of the plants should get to highest because of full-developed rice plants and roots. Transport fractionation at that time is believed to be strongest and a value of −16.7‰ for *ε*_transport_ was measured on D62. At the beginning of the season or aging in the late part of the season, transport fractionation would be relatively weak due to the undeveloped plants with low CH_4_ transport capacity. Therefore, the *ε*_transport_ was found to be −14.7‰ on D37 and −11.1‰ on D98. Many reports have shown a similar variation and it is generally between −16‰ and −11‰^5^,^6^,^7^,^9^,^13^,^19^.

Similar to CH_4_ emission from paddy fields, δ^13^CH_4 (emission)_ is significantly affected by all each process of CH_4_ production, oxidation and transport. At the beginning of the season, the high δ^13^CH_4 (emission)_ was most likely ascribed to the relatively low transport fractionation and the highest *f*_ox_. Subsequently, it became lowest, which was supposed to be the biggest transport fractionation and significantly decreased *f*_ox_. At the late period of the season, the emitted CH_4_ was ^13^C-enriched again, mainly due to an obvious increase of *f*_ac_ at this moment. The δ^13^CH_4 (emission)_ was negatively correlated with CH_4_ emission (Fig. 3b), which further indicates that the higher the CH_4_ flux, the lower the *f*_ox_, thus causing the lower the emitted δ^13^CH_4_. Similar relationships were also observed in the previous studies^9^,^21^,^47^.

In conclusion, this study well showed each process of CH_4_ emission by the measurements of δ^13^CH_4_ from various pools of the paddy field, and found that stable carbon isotope fractionation occurred in CH_4_ production, oxidation and transport. Compared to the roots (1.046–1.080 and 1.013 ± 0.005), *α*_app_ was lower (1.041–1.056) whereas *α*_ox_ was greater (1.021 ± 0.007) in the soil. This suggests that acetate-dependent methanogenesis was more important in paddy soil whereas CH_4_ oxidation was much stronger on the roots. Rice plant-mediated CH_4_ transport fractionation (*ε*_transport_) was found to be −16.7‰ ~ −11.1‰. Temporal variation of CH_4_ emission negatively correlated with δ^13^CH_4 (emission)_ indicates the important relationships of CH_4_ emission with production, oxidation and transport of the CH_4_, which could be demonstrated by the changes of pathway of CH_4_ production and fraction of CH_4_ oxidation. Besides related newly produced δ^13^CH_4_ and finally oxidized δ^13^CH_4_, available carbon fractionation factors were needed to estimate relative contribution of acetate to total CH_4_ production and fraction of CH_4_ oxidized.

## Methods

### Experimental site

The experimental plots are located at Baitu Town, Jurong City, Jiangsu Province, China (31°58′N, 119°18′E). Soil of the field is classified as Typic Haplaquepts, with 11.1 g kg^−1^ in total C, 1.3 g kg^−1^ in total N and −26.8‰ in δ^13^C-value of soil carbon. After wheat was harvested on June 13, 2009, wheat straw with stubble and even wild weeds were all removed from the plots. Then the plots were kept flooded from June 24 to October 15 and drained on October 16 before rice harvest. Seeds of the rice (“*Oryza sativa L*. Huajing 3”) crop were sown into the nursery bed on May 25, seedlings were transplanted into the field on June 26, and the crop was harvested on November 3. Urea was applied at a rate of 300 kg N ha^−1^, 50% as basal fertilizer on June 26, 25% as tillering fertilizer on July 17, and 25% as panicle fertilizer on August 16. Ca(H_2_PO_4_)_2_ (450 kg ha^−1^) and KCL (225 kg ha^−1^) was applied with urea just as basal fertilizer on June 26.

### Field sampling

CH_4_ flux was monitored using the static chamber technique. The flux chambers (0.5 × 0.5 × 1 m), made of plexiglass, covered six hills of rice plants each. Plastic bases for the chambers were installed before rice transplantation in the plots. Removable wooden boardwalks (2 m long) were set up at the beginning of the rice season to avoid soil disturbance during sampling and measuring. To measure CH_4_ flux, gas samples were usually collected once every 4–7 days. Four gas samples from each chamber were collected using 18 mL vacuum vials at 15 min intervals between 09:45 and 10:30 in the morning on each sampling day. To determine carbon isotope composition of the CH_4_ gas (δ^13^CH_4 (emission)_), samples were taken at 15–30 day intervals. Only two gas samples were collected using 0.5 L bags (aluminium foil compound membrane, Delin gas packing Co., Ltd, Dalian, China) with a small battery-driven pump^47^. The first sample was taken after the chamber was closed for 3–5 min, and the second at the end of the 2 h closure period. When CH_4_ flux was monitored, soil temperature at 10 cm depth was simultaneously measured with a hand-carried digital thermometer (Yokogawa Meter and Instruments Corporation, Japan).

Soil pore water samples, 10 cm in depth, were collected using a Rhizon soil moisture sampler (10 RHIZON SMSMOM, Eijkelkamp Agrisearch Equipment, Giesbeek, Netherlands)^47^. The samplers were installed (in triplicate) prior to rice transplanting and then left in the soil throughout the whole season. Samples (about 5 mL) were firstly extracted using 18 mL vacuum vials to flush and purge the sampler before sampling. Then about 10 mL of soil solution was drawn into another vial. Simultaneously, 10 mL of floodwater was collected using a plastic syringe and then transferred in to an 18 mL vacuum vial. Subsequently, all sampling vials were equilibrated by filling in pure N_2_ gas for further analysis with a GC-FID.

CH_4_ emitted via rice plants and the aerenchymatic CH_4_ was measured using specially designed PVC bottomless pots^19^. The pot, 30 cm in height and 17 cm in diameter, was designed to have a water-filled trough around its top, avoiding any possible gas exchange during the sampling times. A PVC plate (18 cm in diameter) with a hole adjustable in diameter to fit the growing plant in the center was placed on top of the pot, allowing the plant to grow through the hole and keeping the plant into two parts. Then, the plant in one pot was cut right above the plate while the plant in the other pot remained intact as control. Finally, chambers (0.3 × 0.3 × 1 m) were laid on the pots, and gas samples in the headspace of the chambers were collected simultaneously with a small battery-driven pump.

Soil cores of the top layer (0–15 cm) were collected at about 15–30-day intervals, and samples of the same plot were first mixed together^48^. Two samples from the mixture, about 50 g each (dry weight), were then taken and transferred promptly into two 250-mL Erlenmeyer flasks separately. Samples in the flasks were prepared into slurries with N_2_-flushed de-ionized sterile water at a soil/water ratio of 1:1. During the whole process, N_2_ was constantly flushed through the samples to remove O_2_ and CH_4_. One flask was sealed for anaerobic incubation. Other flask with air headspace was sealed directly for aerobic incubation. Simultaneously, rice plants together with roots were carefully collected from the plots^48^. The roots were washed clean with N_2_-flushed demineralized water and cut off at 1–2 cm from the root with a razor blade. The fresh roots, about 20 g each portion, were put into flasks, separately, for further preparation and processing in the same way as for the soil samples. All the flasks were sealed with rubber stoppers fitted with silicon septum that allowed sampling of headspace gas. Finally, they were stored under N_2_ at 4 °C and transported back to the lab as soon as possible for further analysis. A small portion of the soil and plant samples were dried for 72 h at 60 °C for determination of isotopic composition of the organic carbon.

### Lab incubations

CH_4_ production potentials were measured under anaerobic incubation. The flasks were flushed with N_2_ consecutively for six times through double-ended needles connecting a vacuum pump to purge the air in the flasks of residual CH_4_ and O_2_. Simultaneously, methanogenesis was determined aerobically using flasks with air headspace directly. They were incubated in darkness at a temperature the same as measured in the field for 50 h. Gas samples were analyzed 1 h and 50 h later after heavily shaking the flasks by hand. CH_4_ production rates were calculated using the linear regression of CH_4_ increasing with the incubation time.

CH_4_ oxidation potentials were determined under aerobic incubation with high concentration of CH_4_ supplemented, using equipment the same as described above. Firstly, pure CH_4_ was injected into each flask to make a high concentration inside (~10,000 *μ*L L^−1^). Then, the flasks were incubated in darkness under the same temperature as measured in the field and shaken at 120 r.p.m. CH_4_ depletion was measured by sampling the headspace gas in the flask after vigorous shaking for subsequent analysis. The first sample was collected generally 30 min after pure CH_4_ was injected. Samples were then taken at 2–3 h intervals during the first 8 h of the experiment. The flasks were left over night and sampled the next day at 2 h intervals again. CH_4_ oxidation rates were calculated by linear regression of CH_4_ depletion with incubation time.

### Chemical measurements

CH_4_ concentrations were analyzed with a gas chromatograph (Shimadzu GC-12A, Kyoto, Japan) equipped with a flame ionization detector. For analysis of carbon isotope composition, the continuous flow technique and a Finnigan MAT 253 isotope ratio mass spectrometer was used (Thermo Finnigan, Bremen, Germany)^47^,^49^. CO_2_ in gas samples was directly analyzed while CH_4_ in gas samples was converted into CO_2_ and separated primarily on a PreCon (pre-concentration device). Then, the gas was piped into a GC equipped with a Pora PLOT Q column (25 m length; 0.35 mm i.d.) at 25 °C under 2.0 × 10^5^ Pa for further separation. The separated gases were finally transferred into the mass spectrometer for δ^13^C determination. The reference and carrier gases used were CO_2_ (99.999% in purity and −23.73‰ in δ^13^C_PDB_-value) and He (99.999% in purity, 20 mL min^−1^), respectively. The precision of the repeated analysis was ±0.2‰ when 2.02 *μ*L L^−1^ CH_4_ was injected. The dried soil and plant samples were analyzed for carbon isotope composition with a Finnigan MAT-251 Isotope Ratio Mass Spectrometer (Thermo Finnigan, Bremen, Germany).

### Statistical analyses

Statistical analysis was done using the SPSS 18.0 software for Windows (SPSS Inc., Chicago). Differences between the four treatments were determined through one-way analysis of variance (ANOVA) and least significant difference (LSD) test. Relationships were assessed using correlation analysis. Significant differences and correlations were set at *P* < 0.05.

## Additional Information

**How to cite this article**: Zhang, G. *et al*. Carbon isotope fractionation reveals distinct process of CH_4_ emission from different compartments of paddy ecosystem. *Sci. Rep.* **6**, 27065; doi: 10.1038/srep27065 (2016).

## Figures and Tables

**Table 1:** CH_4_ production potentials (*μ*g CH_4_ g^−1^ d^−1^), δ^13^C-values (‰) of CH_4_ and CO_2_ in the soil and on the roots under anaerobic incubation, and the corresponding apparent fractionation (*α*_app_) between CO_2_ and CH_4_ calculated by the ratio of (δ^13^CO_2_ + 1000)/(δ^13^CH_4_ + 1000).

**Days after rice transplanting (d)**	**Production**		**δ**^**13**^**CH**_**4**_		**δ**^**13**^**CO**_**2**_		***α***_**app**_	
	**Soil**	**Root**	**Soil**	**Root**	**Soil**	**Root**	**Soil**	**Root**
20	0.13 ± 0.18	3.4 ± 0.7	−71.1 ± 2.4	−69.4 ± 2.8	−18.8 ± 2.9	−15.0 ± 2.3	1.056 ± 0.005	1.058 ± 0.002
50	2.15 ± 0.21	11.1 ± 2.2	−64.4 ± 0.4	−86.9 ± 3.5	−17.0 ± 1.8	−14.1 ± 1.7	1.051 ± 0.002	1.080 ± 0.003
88	0.38 ± 0.12	4.5 ± 0.6	−57.5 ± 1.1	−66.6 ± 2.7	−15.1 ± 0.5	−24.0 ± 2.9	1.045 ± 0.001	1.046 ± 0.005
108	0.22 ± 0.03	3.2 ± 0.9	−53.9 ± 0.2	−72.5 ± 2.9	−14.9 ± 0.4	−23.3 ± 2.8	1.041 ± 0.000	1.053 ± 0.001

**Table 2:** CH_4_ oxidation potentials (*μ*g CH_4_ g^−1^ d^−1^), δ^13^C-values (‰) of CH_4_ at time 0 (δ^13^CH_4 (initial)_) and at time t (δ^13^CH_4 (final)_) in the soil and on the roots under aerobic incubation with high CH_4_ concentration supplemented, and the corresponding CH_4_ oxidation fractionation factor (*α*_ox_) calculated by the Equation (5).

**Days after rice transplanting (d)**	**Oxidation**		**δ**^**13**^**CH**_**4 (initial)**_		**δ**^**13**^**CH**_**4 (final)**_		***α***_**ox**_	
	**Soil**	**Root**	**Soil**	**Root**	**Soil**	**Root**	**Soil**	**Root**
20	4.4 ± 1.4	580 ± 116	−38.4 ± 1.6	−38.7 ± 1.9	−35.6 ± 2.2	−34.0 ± 2.8	1.014 ± 0.002	1.019 ± 0.005
50	6.9 ± 1.1	335 ± 84	−41.0 ± 0.4	−40.4 ± 0.7	−35.0 ± 1.7	−26.5 ± 1.9	1.020 ± 0.002	1.012 ± 0.007
88	5.1 ± 1.9	454 ± 68	−38.7 ± 1.1	−40.5 ± 2.6	−32.5 ± 1.0	−30.6 ± 2.2	1.030 ± 0.004	1.015 ± 0.003
108	2.3 ± 1.3	258 ± 78	−40.3 ± 0.0	−36.0 ± 0.2	−38.4 ± 0.2	−31.2 ± 3.4	1.021 ± 0.009	1.008 ± 0.009

**Table 3:** Relative contribution of acetate to total CH_4_ production (%) in the soil ( *f*_ac_^a^) and on the roots (* f*_ac_^b^).

**Days after rice transplanting (d)**	***f***_**ac**_^**a**^				***f***_**ac**_^**b**^			
	**δ**^**13**^**CH**_**4 (acetate)**_** = **−**37‰**		**δ**^**13**^**CH**_**4 (acetate)**_** = **−**43‰**		**δ**^**13**^**CH**_**4 (acetate)**_** = **−**37‰**		**δ**^**13**^**CH**_**4 (acetate)**_** = **−**43‰**	
	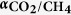 ** = 1.050**	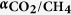 ** = 1.060**	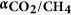 ** = 1.050**	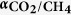 ** = 1.060**	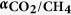 ** = 1.070**	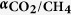 ** = 1.080**	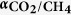 ** = 1.070**	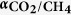 ** = 1.080**
20	−21 ± 4	8 ± 3	−27 ± 4	9 ± 3	24 ± 6	37 ± 7	28 ± 7	41 ± 8
50	−2 ± 6	23 ± 3	−3 ± 7	28 ± 3	−20 ± 1	0 ± 0	−23 ± 2	1 ± 0
88	18 ± 3	39 ± 2	24 ± 4	48 ± 3	42 ± 2	50 ± 15	47 ± 2	56 ± 17
108	32 ± 0	50 ± 0	42 ± 0	61 ± 0	29 ± 0	39 ± 11	33 ± 0	44 ± 12

*f*_ac_^a^ and *f*_ac_^b^ was calculated with Equation (2) using δ^13^C-values of CH_4_ anaerobically produced in the soil and on the roots (Table 1) as originally produced δ^13^CH_4_, respectively.

**Table 4:** Fraction of CH_4_ oxidized (%) in the rhizosphere ( *f*_ox_^a^) and at the soil-water interface ( *f*_ox_^b^) in field conditions, and at the surfaces of soil ( *f*_ox_^c^) and rice roots ( *f*_ox_^d^) in lab conditions.

**Days after rice transplanting (d)**	***α***_**ox**_** = 1.021**			***α***_**ox**_** = 1.013**	***α***_**ox**_** = 1.038**			
	***f***_**ox**_^**a**^	***f***_**ox**_^**b**^	***f***_**ox**_^**c**^	***f***_**ox**_^**d**^	***f***_**ox**_^**a**^	***f***_**ox**_^**b**^	***f***_**ox**_^**c**^	***f***_**ox**_^**d**^
20	108 ± 16	88 ± 16	78 ± 18	235 ± 26	61 ± 9	49 ± 7	44 ± 10	82 ± 9
50	51 ± 6	116 ± 25	61 ± 6	372 ± 43	29 ± 3	65 ± 14	34 ± 3	130 ± 16
88	42 ± 16	86 ± 22	27 ± 3	209 ± 27	23 ± 9	48 ± 12	15 ± 2	73 ± 10
108	33 ± 22	84 ± 12	−4 ± 13	244 ± 24	19 ± 12	47 ± 7	−2 ± 8	86 ± 9

*f*_ox_^a^ was calculated with Equation (5) using δ^13^C-values of CH_4_ anaerobically produced in the soil (Table 1) as δ^13^CH_4 (original)_ and δ^13^CH_4 (emission)_ (Fig. 3a) minus −14.2‰ as δ^13^CH_4 (oxidized)_;

*f*_ox_^b^ was calculated with Equation (5) using δ^13^C-values of CH_4_ in soil pore water (Fig. 2) as δ^13^CH_4 (original)_, and δ^13^C-values of CH_4_ in floodwater (Fig. 2) as δ^13^CH_4 (oxidized)_;

*f*_ox_^c^ and *f*_ox_^d^ were calculated with Equation (5) using δ^13^C-values of CH_4_ anaerobically produced in the soil and on the roots (Table 1) as δ^13^CH_4 (original)_ and δ^13^C-values of CH_4_ aerobically produced in the soil and on the roots (Fig. 1) as δ^13^CH_4 (oxidized)_, respectively.
